# Supplementary material for: A national survey on current clinical practice pattern of Korean Medicine doctors for treating obesity
Source: PLoS One. 2022 Mar 24;17(3):e0266034. doi: 10.1371/journal.pone.0266034 (PMC8947078; doi:10.1371/journal.pone.0266034)
Supplement: S1 Table — (DOCX) [file pone.0266034.s001.docx]

**S1 Table.** **Patient satisfaction with treatment for obesity.**

|  |  | **Total**  **(n = 1084)** | **Specialized in obesity treatment** | |
| --- | --- | --- | --- | --- |
|  |  |  | **Specialized**  **(n = 51)** | **Non-specialized**  **(n = 1033)** |
| Patient satisfaction with treatment for obesity | |  |  |  |
|  | Herbal medicine | 986 (91.0) | 51 (100.0) | 935 (90.5) |
|  | Control diet (e.g. fasting, calorie restriction) | 25 (2.3) | 0 | 25 (2.4) |
|  | Electroacupuncture | 22 (2.0) | 0 | 22 (2.1) |
|  | Lifestyle intervention for obesity | 12 (1.1) | 0 | 12 (1.2) |
|  | Moxibustion | 9 (0.8) | 0 | 9 (0.9) |
|  | Acupuncture | 8 (0.7) | 0 | 8 (0.8) |
|  | Chuna | 8 (0.7) | 0 | 8 (0.8) |
|  | Pharmacoacupuncture | 7 (0.7) | 0 | 7 (0.7) |
|  | Cupping | 4 (0.4) | 0 | 4 (0.4) |
|  | Qigong | 1 (0.1) | 0 | 1 (0.1) |
|  | Others | 2 (0.2) | 0 | 2 (0.2) |

All data are express in N (%). KM: Korean medicine.
