# Supplementary material for: A national survey on current clinical practice pattern of Korean Medicine doctors for treating obesity
Source: PLoS One. 2022 Mar 24;17(3):e0266034. doi: 10.1371/journal.pone.0266034 (PMC8947078; doi:10.1371/journal.pone.0266034)
Supplement: S2 Table — (DOCX) [file pone.0266034.s002.docx]

**S2 Table. Difficulties in obesity treatment.**

|  |  | **Total**  **(n = 1084)** | **Specialized in obesity treatment** | |
| --- | --- | --- | --- | --- |
|  |  |  | **Specialized**  **(n = 51)** | **Non-specialized**  **(n = 1033)** |
| Difficulties in obesity treatment^†^ | |  |  |  |
|  | Financial burden of patient | 636 (58.7) | 18 (35.3) | 618 (59.8) |
|  | Low treatment effects compared to cost | 191 (17.6) | 6 (11.8) | 185 (17.9) |
|  | Drug interactions with western medication | 180 (16.6) | 12 (23.5) | 168 (16.3) |
|  | Concern to side effects | 156 (14.4) | 8 (15.7) | 148 (14.3 |
|  | Absence of KM standard clinical protocol for obesity | 153 (14.1) | 0 | 153 (14.8) |
|  | Low preference by patients | 113 (10.4) | 4 (7.8) | 109 (10.6) |
|  | Difficulties in performing treatment methods | 95 (8.8) | 2 (3.9) | 93 (9.0) |
|  | No difficulties | 114 (10.5) | 14 (27.5) | 100 (9.7) |
|  | Others | 57 (5.3) | 1 (2.0) | 56 (5.4)) |

All data are express in N (%). KM: Korean medicine. ^†^Multiple responses allowed.
