# Supplementary material for: A national survey on current clinical practice pattern of Korean Medicine doctors for treating obesity
Source: PLoS One. 2022 Mar 24;17(3):e0266034. doi: 10.1371/journal.pone.0266034 (PMC8947078; doi:10.1371/journal.pone.0266034)
Supplement: S1 Appendix — (PDF) [file pone.0266034.s003.pdf]

Appendix

Questionnaire: A national survey on current clinical practice pattern of Korean Medicine doctors for treating obesity.

This survey is conducted for the purpose of investigating the current clinical practice status of Korean medicine treatment for obesity. You can voluntary participate and can also discontinue at any time during survey. As the survey is in the form of an online survey, there is no risk of participating in the survey. Please select the appropriate answer for each question based on your clinical experience. The results of this survey can be published in domestic or overseas academic presentations and related journals.

Do you understand the purpose of this survey and voluntarily participate in this survey?

① YES ② NO

[Demographic information of participant]

1. What is your sex?

① male ② female

2. What is your age (years old)?

① ≤ 29 ② 30 ~ 39 ③ 40 ~ 49

④ 50 ~ 59 ⑤ 60 ≤

3. How many years have you been in clinical practice (years)?

① < 5 ② 5 ~ 9 ③ 10 ~ 14

④ 15 ~ 19 ⑤ 20 ~ 29 ⑥ 30 ≤

4. What is your affiliated institution?

① Korean medicine clinic (general)

② Korean medicine clinic (obesity treatment specialized)

③ Korean medicine hospital

④ Public hospital

⑤ Public community health center

⑥ Convalescent/Geriatric hospital

⑦ Others

5. Where is your affiliated institution located?

① Seoul ② Busan/Daegu/Ulsan/Gyeongsang

③ Incheon/Gyeonggi/Gangwon ④ Daejeon/Sejong/Chungcheong

⑤ Gwangju/Jeolla/Jeju

6. What is your highest academic degree?

① Bachelor ② Master ③ Ph. D.

7. Are you specialist in Korean medicine?

① Yes (specialist) ② No (general practitioner)

7-1. Please choose your specialty in Korean medicine.

① Internal medicine ② Gynecology ③ Pediatric ④ Neuropsychiatry

⑤ Otolaryngology and Dermatology ⑥ Rehabilitation

⑦ Acupuncture and moxibustion medicine ⑧ Sasang constitutional medicine

[Current state of clinical practice for obesity]

8. How many years have you been treating obesity (free text)? _______years

9. How many patients do you treat for obesity (free text)? _______patients (per year)

10. What is the primary gender of patients you treat for obesity?

① Male ② Female

10. What is the main age group of patients you treat for obesity?

① ≤ 19 ② 20 ~ 29 ③ 30 ~ 39

④ 40 ~ 49 ⑤ 50 ~ 59 ⑥ 60 ≤

11. What is the main obesity level of patients you treat for obesity?

① Underweight (BMI < 18.5) ② Normal weight (18.5 ≤ BMI ≤ 22.9)

③ Overweight (23 ≤ BMI ≤ 24.9) ④ Obesity Class I (25 ≤ BMI ≤ 29.9)

⑤ Obesity Class II (30 ≤ BMI ≤ 34.9) ⑥ Obesity Class III (35 ≤ BMI)

12. What is the main comorbidity of patients? (multiple choice allowed)

① Metabolic syndrome ② Hypertension ③ Diabetes

④ Digestive diseases ⑤ Dyslipidemia ⑥ Arthritis

⑦ Cardiovascular diseases ⑧ Others ⑨ No comorbidities

13. How long on average duration do you treat patients for obesity?

① under 1 week ② 1 to 2 weeks ③ 2weeks to 1 month

④ 1 to 3 months ⑤ 3 to 6 months ⑥ 6 months to 1 year

⑦ over 1 year

14. How often do patients being treated for obesity visit (free number)? ________ per month

15. How long do you treat on average time to patients for obesity per visit?

① less than 10 minutes ② 10 to 20 minutes

③ 20 to 30 minutes ④ more than 30 minutes

16. Do you provide management care to patients after taking herbal medicine for weight loss?

① Yes (go to Q. 16-1) ② No (go to Q. 17)

16-1. How long do you provide management care to patients?

① under 2 weeks ② 2weeks to 1 month ③ 1 to 3 months

④ 3 to 6 months ⑤ over 6 months

17. What is the average weight loss of patients by treatment? (Please, fill in both of below)

- **________% of Body weight**
- **Average _______Kg**

[Diagnosis and intervention for obesity]

18. What do you use diagnostic tool or device for obesity? (multiple choice allowed)

① Pulse diagnosis instrument ② Body thermometer

③ Body composition analyzer ④ Ryodoraku analyzer

⑤ KM Syndrome Differentiation Questionnaire for Obesity

⑥ Tongue diagnosis instrument ⑦ Others ⑧ None

19. What do you use KM syndrome differentiation for obesity treatment?

① Yes (go to Q.19-1) ② No (Using routine prescription)

19-1. Which KM syndrome differentiation you use for obesity treatment? (multiple choice allowed)

① Eight principle pattern identification ② Organ system diagnosis

③ Defensive Qi and nutrient blood diagnosis

④ Sasang constitutional medicine diagnosis

⑤ Meridian system diagnosis ⑥ Six meridian diagnosis

⑦ Diagnostic type by the KM obesity clinical practice guideline ⑧ Others

20. What is the primary indicator for evaluating obesity treatment? (multiple choice allowed)

① Body weight ② Body Mass Index (BMI)

③ Percentage of body fat ④ Abdominal fat rate ⑤ Waist circumstance

21. What is the primary determinant for therapeutic intervention? (multiple choice allowed)

① Lifestyle habits (e.g. exercise, eating habits and nutritional status)

② Obesity level (e.g. body weight, BMI)

③ Purposes of treatment (e.g. weight loss, body shape)

④ Medical history or comorbidities

⑤ Age

⑥ Estimated duration of treatment

⑦ Sasang constitution type

⑧ Economic factor

22. What is the primary intervention do you use for obesity treatment? (multiple choice allowed)

① Herbal medicine ② Acupuncture (including ear acupuncture)

③ Electroacupuncture ④ Pharmacoacupuncture

⑤ Moxibustion ⑥ Cupping ⑦ Chuna

⑧ Control diet (e.g. fasting, caloric restriction) ⑨ Qigong

⑩ Lifesytle intervention for obesity ⑪ Others

22. What is commonly prescribed herbal formula for obesity? (multiple choice allowed)

① Taeeumjowui-tang ② Bangpungtongseong-san (Bofutsushosan)

③ Euiiyin-tang ④ Bangkihwangki-tang (Boiogito)

⑤ Cheongpyesagan-tang ⑥ Jowiseungcheung-tang

⑦ Buhnsimgieum ⑧ Gambi-hwan

⑨ Gamrosu ⑩ Anmyungambi-tang ⑪ Others

23. How long is the average duration of herbal medicine administration? ___________weeks

24. Do you have any herbs frequently prescribe regardless of KM syndrome differentiation or herbal formula?

① Yes (go to Q.24-1) ② No (go to Q.25)

24-1. Please write herb frequently prescribed regardless of KM syndrome differentiation or herbal formula. (free text)

____

25. Do you prescribe Ephedrae Herba for obesity treatment?

① Yes (go to Q.26) ② No (go to Q.29)

26. What is the average prescribed dose of Ephedrae Herba for obesity treatment?

- **Minimum dose __________ g/day**
- **Maximum dose __________ g/day**

27. What is the primary factor for deciding the dose of Ephedrae Herba for obesity treatment? (multiple choice allowed)

① Obesity ② Sasang constitutional type

③ Sleeping habits ④ Caffeine sensitivity (e.g. Heart palpitation)

⑤ Others

28. Have you ever had a patient who was prescribed Ephedrae Herba complained of uncomfortable symptoms?

① Yes (go to Q.28-1) ② No (go to Q.29)

28-1. Which was the uncomfortable symptoms complained by the patient who was prescribed Ephedrae Herba? (multiple choice allowed)

① Abnormal level on blood test (e.g. ALT, AST, BUN, Creatinine)

② Cardiovascular events (e.g. palpitation, hypertension, tachycardia)

③ Neuropsychiatric events (e.g. anxiety, insomnia)

④ Gastrointestinal events (e.g. nausea, vomiting, indigestion)

⑤ Dermatological events

⑥ Musculoskeletal events

⑦ Respiratory events

⑧ Geniourinary events (e.g. frequent urination, irregular menstruation)

⑨ Severe adverse events (e.g. hospitalization, heart attack or death)

⑩ Others

28-2. Do you notify caution for caffeine consumption to patient who is prescribed Ephedrae Herba?

① Yes ② No

29. Have you ever had a patient who was prescribed herbal medicine not containing Ephedrae Herba complained of uncomfortable symptoms?

① Yes (go to Q.29-1) ② No

29-1. Which was the uncomfortable symptoms complained by the patient who was prescribed herbal medicine not containing Ephedrae Herba? (multiple choice allowed)

① Abnormal level on blood test (e.g. ALT, AST, BUN, Creatinine)

② Cardiovascular events (e.g. palpitation, hypertension, tachycardia)

③ Neuropsychiatric events (e.g. anxiety, insomnia)

④ Gastrointestinal events (e.g. nausea, vomiting, indigestion)

⑤ Dermatological events (e.g. rash, itching)

⑥ Musculoskeletal events

⑦ Respiratory events

⑧ Geniourinary events (e.g. frequent urination, irregular menstruation)

⑨ Severe adverse events (e.g. hospitalization, heart attack or death)

⑩ Others

30. Which intervention has the highest patient satisfaction?

① Herbal medicine ② Acupuncture (including ear acupuncture)

③ Electroacupuncture ④ Pharmacoacupuncture

⑤ Moxibustion ⑥ Cupping ⑦ Chuna

⑧ Control diet (e.g. fasting, caloric restriction) ⑨ Qigong

⑩ Lifesytle intervention for obesity ⑪ Others

31. What is the difficulty in obesity treatment?

① Difficulties in performing treatment methods

② Financial burden of patient

③ Concern to side effects

④ Low treatment effects compared to cost

⑤ Low preference by patients

⑥ Absence of KM standard clinical protocol

⑦ Drug interaction with western medication

⑧ Others

⑨ No difficulties

Thanks for participating.
